# There might be blood: a scoping review on women’s responses to contraceptive-induced menstrual bleeding changes

**DOI:** 10.1186/s12978-018-0561-0

**Published:** 2018-06-26

**Authors:** Chelsea B. Polis, Rubina Hussain, Amanda Berry

**Affiliations:** Guttmacher Institute, 125 Maiden Lane, 7th Floor, New York, NY 10038 USA

**Keywords:** Contraception, Menstruation, Menstrual bleeding changes, Contraceptive non-use and discontinuation, Side effects, Health concerns, Amenorrhea

## Abstract

**Introduction:**

Concern about side effects and health issues are common reasons for contraceptive non-use or discontinuation. Contraceptive-induced menstrual bleeding changes (CIMBCs) are linked to these concerns. Research on women’s responses to CIMBCs has not been mapped or summarized in a systematic scoping review.

**Methods:**

We conducted a systematic scoping review of data on women’s responses to CIMBCs in peer-reviewed, English-language publications in the last 15 years. Investigator dyads abstracted information from relevant studies on pre-specified and emergent themes using a standardized form. We held an expert consultation to obtain critical input. We provide recommendations for researchers, contraceptive counselors, and product developers.

**Results:**

We identified 100 relevant studies. All world regions were represented (except Antarctica), including Africa (11%), the Americas (32%), Asia (7%), Europe (20%), and Oceania (6%). We summarize findings pertinent to five thematic areas: women’s responses to contraceptive-induced non-standard bleeding patterns; CIMBCs influence on non-use, dissatisfaction or discontinuation; conceptual linkages between CIMBCs and health; women’s responses to menstrual suppression; and other emergent themes. Women’s preferences for non-monthly bleeding patterns ranged widely, though amenorrhea appears most acceptable in the Americas and Europe. Multiple studies reported CIMBCs as top reasons for contraceptive dissatisfaction and discontinuation; others suggested disruption of regular bleeding patterns was associated with non-use. CIMBCs in some contexts were perceived as linked with a wide range of health concerns; e.g., some women perceived amenorrhea to cause a buildup of “dirty” or “blocked” blood, in turn perceived as causing blood clots, fibroids, emotional disturbances, weight gain, infertility, or death. Multiple studies addressed how CIMBCs (or menstruation) impacted daily activities, including participation in domestic, work, school, sports, or religious life; sexual or emotional relationships; and other domains.

**Conclusions:**

Substantial variability exists around how women respond to CIMBCs; these responses are shaped by individual and social influences. Despite variation in responses across contexts and sub-populations, CIMBCs can impact multiple aspects of women’s lives. Women’s responses to CIMBCs should be recognized as a key issue in contraceptive research, counseling, and product development, but may be underappreciated, despite likely – and potentially substantial – impacts on contraceptive discontinuation and unmet need for modern contraception.

**Electronic supplementary material:**

The online version of this article (10.1186/s12978-018-0561-0) contains supplementary material, which is available to authorized users.

## Plain English summary

Some contraceptive methods cause changes in women’s menstrual bleeding patterns. For example, a woman’s period may become lighter or heavier, longer or shorter, less regular, or may disappear altogether. Concerns about side effects and health issues – including those related to changes to menstrual bleeding patterns – may limit use of contraceptive methods. However, the research on how women respond to contraceptive-induced menstrual bleeding changes (CIMBCs) has not been summarized in a systematic scoping review. We collected and summarized the body of evidence on women’s responses to CIMBCs in a standardized manner. We identified 100 studies from around the world relevant to this issue. We summarized what studies found regarding how women respond when contraceptive methods stop their periods or cause other non-standard bleeding patterns, and the extent to which CIMBCs make women unhappy with their method of contraception, or stop their method of contraception, or not use any method of contraception. We also summarized what the evidence suggests regarding how women think about CIMBCs in terms of their own health, as well as other themes that emerged from our review of studies. While women across countries and populations respond differently to different CIMBCs, due to individual and social influences, it is clear that CIMBCs impact many areas of women’s lives. It is important that researchers, medical providers, and contraceptive product developers recognize this as an important issue, and we offer recommendations on how to do so.

## Background

About 99 million unintended pregnancies occur annually, the majority of which could be prevented through use of modern contraception [1, 2]. Concerns about side effects and health issues are common reasons for non-use or discontinuation of contraception among women who do not desire pregnancy [3–5]. Among married women with an unmet need for contraception in 52 developing countries, 7–53% reported not using a method due to these concerns [3]. Some smaller (often qualitative) studies report on women’s experiences with or fears about side effects or health concerns in relation to various contraceptive methods, but few large or nationally-representative studies specifically investigate these issues in detail [6]. Some large-scale surveys (e.g., PMA2020 and Demographic and Health Surveys (DHS)) ask about reasons for contraceptive non-use and discontinuation, and include health concerns, fear of side effects, and interference with bodily processes as broad response categories, but neither survey asks which specific side effects or health concerns led to non-use or discontinuation [7, 8]. Furthermore, other broad response categories, such as self or partner opposition to contraceptive use, inconvenience of use, or other reasons, may be intertwined with health or side effect-related concerns. Therefore, it is difficult to estimate the prevalence or impact of these concerns, or to disentangle which issues are of greatest concern to women or couples, particularly on a national scale.

Furthermore, while certain contraceptive side effects are clinically documented, various contraceptive-induced bodily processes may be interpreted variably by different individuals. Perceptions of contraceptive-related side effects may be rooted in personal experience, knowledge of others’ experiences, or misinformation [9, 10]. While discordance between documented and perceived side effects is acknowledged in the literature [11, 12], both experienced and perceived side effects can be highly influential in contraceptive decision-making processes [10, 13]. Furthermore, cultural norms and values may shape tolerance (or lack thereof) and fears around various side effects.

Hormonal contraceptive methods and IUDs may induce changes in menstrual bleeding patterns [14–16], which can impact willingness to try or continue using these methods, or method satisfaction [6, 17–23]. Contraceptive-induced menstrual bleeding changes (CIMBCs) may include bleeding patterns which are predictable but diverge from a “typical” menstrual pattern (such as amenorrhea, commonly induced by methods such as progestin-only injectables, or heavy, prolonged bleeding often experienced by copper IUD users [24, 25]), or may cause unpredictable bleeding patterns. While menstrual bleeding can be measured in straightforward clinical categories, there may be large ranges defined around normal menstruation [26, 27] and these clinical definitions may not be in line with women’s perceptions of normal bleeding. Furthermore, women may experience CIMBCs they consider abnormal or unacceptable, but may still clinically fall within the range of normal.

In addition to inconvenience (for unpredictable bleeding patterns in particular), and the menstrual hygiene management costs of many bleeding patterns, some individuals may perceive changes to bleeding patterns as being tied to overall notions about their health [23, 28, 29] or to physical or mental health issues [6, 9, 10, 12, 13, 20, 23, 29]. For example, some women fear that injectable-induced amenorrhea leads to permanent infertility, which is not supported in the literature [30]. Counseling may not always be comprehensive enough to adequately prepare women to fully understand, anticipate, or manage CIMBCs [31]. Though difficult to precisely quantify (owing in part to lack of sufficiently specific nationally representative data, as described above), some evidence suggests that CIMBCs are a central aspect of what women mean when they report “side effects” or “health concerns” [32–35], and may be an important reason for non-use or discontinuation. However, the importance of CIMBCs may be underappreciated in the reproductive health field as a key contributor to issues such as unmet need for modern contraception.

In sum, side effects constitute a major reason for contraceptive non-use and discontinuation, and CIMBCs are linked, in both real and perceived ways, with a range of concerns. Differences exist between what bleeding patterns a woman *prefers* (including the potential for no bleeding changes) and what she is willing to *tolerate* in exchange for the benefits of the contraceptive options available to her [36]. Understanding women’s responses (including attitudes and behaviors) to experienced or anticipated CIMBCs has significant implications for current contraceptive use patterns and for the development of future products, including contraceptives and contraceptive-containing multipurpose prevention technologies (MPTs), which are products in development that aim to deliver varied combinations of contraception and prevention from HIV and other STIs. However, to our knowledge, no recent systematic scoping reviews have examined the extent and range of research on this topic. Thus, we conducted a scoping review to gather and synthesize data on women’s responses to CIMBCs and to provide recommendations for providers, researchers, and product developers.

## Methods

### Methodological approach

Scoping reviews are defined as “a form of knowledge synthesis that addresses an exploratory research question aimed at mapping key concepts, types of evidence, and gaps in research related to a defined area or field by systematically searching, selecting, and synthesizing existing knowledge” [37]. Whereas systematic reviews typically focus on a well-defined question of interest (for which appropriate study designs can be identified in advance), scoping reviews are suitable for broader areas of inquiry, for which multiple study designs may be relevant [38]. Women’s responses to anticipated or experienced CIMBCs have been assessed in clinical trials, surveys, qualitative studies, and other designs. We aimed to systematically search the literature for relevant content, to organize this information by summarizing the research questions addressed and articulating key themes, and to identify gaps in the existing literature. While we refer to countries in which studies were conducted, most studies were not nationally representative, so findings are not necessarily nationally generalizable.

### Search strategy

We sought to identify peer-reviewed, English-language publications focused on women’s responses to CIMBCs among women of reproductive age in any country, published in peer-reviewed journals within the last 15 years (since norms may change over time) [39]. We searched PubMed using Medical Subject Headings (MeSH terms) as follows: (“Menstruation/psychology”[MeSH Terms] OR (“Contraceptive Agents, Female”[MeSH Terms] AND (“menstruation”[MeSH Terms] OR “Menstruation Disturbances”[MeSH Terms] OR “Metrorrhagia”[MeSH Terms]))) AND ((“2002/01/01”[PDAT]: “2017/03/14”[PDAT]) AND “humans”[MeSH Terms] AND English[lang] AND “female”[MeSH Terms]). We also reviewed reference lists of included studies and consulted with topical experts to identify any additional uncaptured studies. We did not search the grey literature.

### Inclusion criteria

To maximize comprehensiveness and feasibility, while minimizing inclusion of irrelevant or minimally informative studies, we required that included studies made reference to examining women’s responses to CIMBCs in the title and/or abstract. We excluded studies examining CIMBCs without assessing women’s responses to those changes, and those addressing several other narrow topical areas, including:

- Studies that did not explicitly examine women’s responses with respect to CIMBCs (e.g., studies on attitudes, cultural beliefs, or practices related to menstruation; age of menarche; impacts of factors such as stress on menstrual patterns; menstrual hygiene management; menstrual synchrony; etc.),
- Studies addressing specific menstrual issues, or intersections of menstruation with specific medical issues (e.g., dysmenorrhea, pre-menstrual syndrome, oral contraceptive-induced menstrual migraine, various psychological conditions, etc.) or constructs (e.g., menstruation and body image),
- Studies conducted within highly specific sub-populations (e.g., women in the military, women with intellectual disabilities), or studies pertinent to methods of contraception that are not typically used as ongoing methods (e.g., emergency contraception),
- Clinical guidance or reviews, or counseling/prescribing habits of physicians (e.g., as it relates to medically induced amenorrhea).

### Study screening and data abstraction

One author (CBP) conducted the initial title/abstract screening using Covidence (advancing abstracts to full-text review in the event of uncertainty) [40], and two investigators (dyads of CBP, RH, and/or AB) read remaining full texts to determine inclusion and abstract data. We developed an abstraction form and pilot tested it on multiple studies to refine it. We collected information about the study setting, population, and methodology, including whether it assessed a particular contraceptive method or was non-specific. As scoping reviews generally do not assess study quality [41], these details were minimal. In addition to examining the geographic distribution of identified studies, we extracted information about four key questions (1–4 below), and additional pertinent themes that we mutually identified as emerging from the literature:

1. Women’s responses related to contraceptive-induced amenorrhea or other non-standard bleeding frequencies
2. CIMBCs as a reason for non-use, discontinuation, or dissatisfaction
3. Conceptual linkages between CIMBCs and health risks or side effects
4. Use of contraception for deliberate menstrual suppression
5. Other emergent themes

Since bleeding changes occurring from menstrual suppression are deliberately induced, rather than incidental to use of the method, we mention these findings only briefly, but incorporate them where relevant to other themes. Since we excluded studies on highly specific sub-populations, our findings regarding attitudes toward menstrual suppression are not representative of specific subpopulations (e.g., women in the military, women with intellectual disabilities) that may have significantly different attitudes toward menstrual suppression.

### Expert consultation

To enhance the utility and rigor of our review [37, 38, 42], we discussed our preliminary findings in a consultation with five experts on contraceptive acceptability, clinical or social research on CIMBCs, clinical contraceptive provision, and contraceptive and/or MPT product development. We obtained feedback on our overall approach, our literature search methods, presentation of results, and how to make the paper most useful for providers, researchers, and product developers.

## Results

### Overview of included studies

**Fig. 1:**
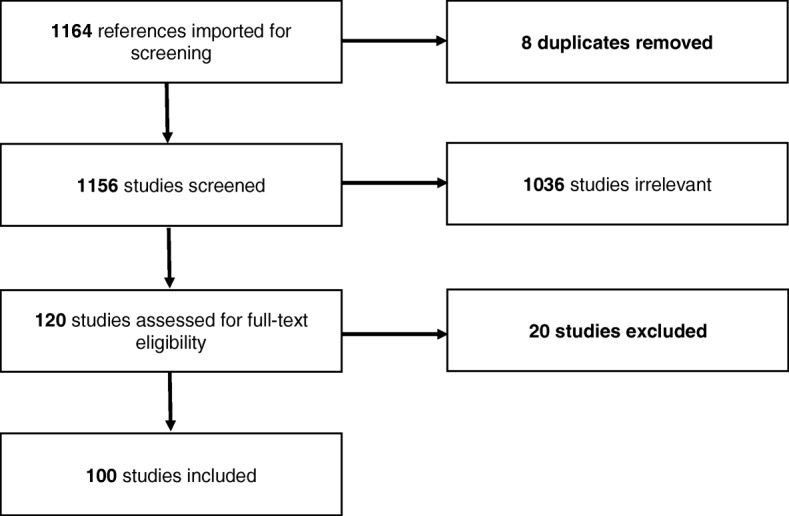
Study flowchart

Of 1164 references identified, 100 were considered appropriate for inclusion (Fig. 1). All geographic world regions were represented (except Antarctica), including studies in Africa (11%), the Americas (32%), Asia (7%), Europe (20%), and Oceania (6%) (Table 1). The remaining studies (24%) were multi-country studies or systematic reviews. Publication dates ranged from 2002 to 2016.

**Table 1:** Geographic representation of included studies

	N	% of included studies across and within subregions	Countries represented (and number of studies within that country)	References
Africa	11	11%		
Northern Africa	1	9%	Egypt (1)	[115]
Eastern Africa	2	18%	Kenya (2)	[107, 132]
Middle Africa	0	0%	–	–
Southern Africa	2	18%	South Africa (2)	[47, 122]
Western Africa	6	55%	Nigeria (3), Mali (1), Ghana (2)	[43, 76, 87, 94, 102, 104]
Americas	32	32%		
Latin America and the Caribbean	10	31%	Dominican Republic (1), Mexico (3), Brazil (6)	[44, 57, 68, 78, 81, 84, 95, 103, 124, 137]
Northern America	22	69%	US (18), Canada (3), Unspecified (1)	[46, 58, 59, 61, 62, 71, 75, 85, 86, 90–92, 111, 112, 125–127, 130, 133–136]
Antarctica	0	na	–	–
Asia	7	7%		
Central Asia	0	0%	–	–
Eastern Asia	1	14%	China (1)	[64]
Southeastern Asia	1	14%	Thailand (1)	[114]
Southern Asia	3	43%	India (1), Bangladesh (1), Iran (1)	[63, 83, 99]
Western Asia	2	29%	Turkey (2)	[45, 123]
Europe	20	20%		
Eastern Europe	0	0%	–	–
Northern Europe	10	50%	Finland (1), Ireland (1), Netherlands (1), UK (7)	[51, 52, 82, 97, 98, 100, 105, 109, 113, 145]
Southern Europe	6	30%	Italy (3), Spain (3)	[66, 67, 77, 79, 88, 138]
Western Europe	4	20%	Austria (2), Germany (1), Switzerland (1)	[48, 53, 60, 110]
Oceania	6	6%		
Australia and New Zealand	6	100%	Australia (5), New Zealand (1)	[54, 96, 106, 121, 139, 141]
Melanesia, Micronesia, Polynesia	0	0%		–
Multi-country studies or systematic reviews	24	24%	Australia, Austria, Belgium, Brazil, Canada, Chile, China, Czech Republic, Dominican Republic, Finland, France, Germany, Hungary, Indonesia, Israel, Italy, Japan, Kenya, Netherlands, New Zealand, Nigeria, Norway, Poland, Russia, Scotland, Slovakia, South Africa, Spain, Sweden, Switzerland, Thailand, Tunisia, Turkey, Ukraine, UK, US, Zimbabwe; unspecified countries in Europe, Asia, and Western Europe; countries included in studies in systematic reviews	[49, 50, 55, 56, 65, 69, 70, 72–74, 80, 89, 93, 101, 108, 116–120, 128, 131, 140, 160]

Cross-sectional survey designs were most common (32%), followed by longitudinal studies including RCTs (30%), qualitative studies (19%), retrospective chart reviews (12%), systematic reviews (6%), and mixed method studies (1%). Inclusion criteria varied across studies, though some assessed sub-populations (e.g., women choosing or discontinuing a particular contraceptive method, adolescents or young women, women living with HIV, etc.) Some studies did not limit their focus to specific contraceptive methods (31%); the remainder focused on implants (23%), IUDs (12%), OCPs (14%), injectables (4%), the vaginal ring (2%), or multiple specific methods (14%).

CIMBCs as a reason for non-use, discontinuation, and dissatisfaction were the most commonly explored themes (71 studies), followed by women’s attitudes specifically towards contraceptive-induced amenorrhea or other non-standard bleeding frequencies (33) and conceptual linkages between CIMBCs and health risks and side effects (33). The use of contraception for menstrual suppression was explored, in varying depth, in 28 studies. We summarized additional key themes stemming from 41 studies.

### Women’s responses related to contraceptive-induced amenorrhea and other non-standard bleeding frequencies

Women’s responses varied substantially across individuals, communities, and regions. In some studies, amenorrhea was primarily viewed negatively [43–50]. In addition to health concerns (detailed below), many women were generally suspicious of amenorrhea [44], saw it as a disadvantage of hormonal contraception [45–50], and identified menstruation as a natural state of womanhood [44, 45]. More positive views of amenorrhea emerged in some studies [48–54], mainly centering around convenience [44, 50, 51, 55] or avoidance of menstruation-associated problems (e.g., painful periods) [47, 55].

Across included surveys, women’s preference for amenorrhea ranged between 0% [56] (in Tunisia) and 65% [57] (in Brazil) (Table 2) [53, 55–71]. Preferences for regular, non-monthly menstrual cycles (i.e., various durations of longer than one month but less than one year) ranged between 0% [56] (in Indonesia) and 66% [68] (in Mexico). Generally, amenorrhea appears more commonly preferred in North America, Europe and South America, whereas trends for other bleeding pattern preferences are less prominent (Table 2). It is important to note that over half of studies examining women’s bleeding pattern preferences were conducted in North America or Europe, and that these findings may not generalize to other contexts.

**Table 2:** Studies presenting women’s preferences for amenorrhea or other bleeding patterns by percentage of women

Region of study	Country of study	Author	Publication Year	Study Population	Percentage of Women Preferring bleeding patterns		
					Amenorrhea/Never Bleeding	Other than monthly bleeding or amenorrhea (ranging from 2 months to 1 year)	Monthly Bleeding
Africa	Nigeria	Glasier	2003	Aged 20+	Sagamu: 13%	Sagamu: 12%	Sagamu: 71%
	South Africa	Glasier	2003	Aged 20+	Cape Town: 9–36%^a^	Cape Town: 15–27%^a^	Cape Town: 30–49%^a^
	Tunisia	d’Arcangues	2011	Aged 18–38 Norplant users	Tunis: 0%	Tunis: 5%	Tunis: 95%
Americas	Brazil	Snow	2007	Aged 18–49	~ 33%^b^	~ 37%^b^	~ 25%^b^
	Brazil	Makuch	2012	Aged 18–39	65.3%	18.2%	13.5%
	Brazil	Szarewski	2012	Aged 15–49^c^	16%	55%	n/a
	Canada	Nguyen	2011	Ages not specified	56%^b^	n/a	n/a
	Canada	Szarewski	2012	Aged 15–49^c^	15%	38%	n/a
	Chile	d’Arcangues	2011	Aged 18–38 Norplant users	Santiago: 40%	Santiago: 13%	Santiago: 47%
	Dominican Republic	d’Arcangues	2011	Aged 18–38 Norplant users	Santo Domingo: 14%	Santo Domingo: 2%	Santo Domingo: 84%
	Mexico	Marvan	2009	Aged 20–25 and 40–50 Women aged 20–25 were college students	n/a	66%	34%
	United States	Andrist (Contraception)	2004	Aged 18–40	59%		n/a
	United States	Andrist (JAANP)	2004	Aged 18–40	57%		n/a
	United States	Edelman	2007	Mean age 27	38%	29%	34%
	United States	Snow	2007	Aged 18–49	~ 33%^b^	~ 45%^b^	~ 16%^b^
	United States	Szarewski	2012	Aged 15–49^c^	16%	49%	n/a
	United States	Lakehomer	2013	Mean age 21.4 College students Recent or current contraceptive use	28%	65%	n/a
Asia	China	Glasier	2003	Aged 20+	Hong Kong: 6% Shanghai: 15%	Hong Kong: 39% Shanghai: 30%	Hong Kong: 42% Shanghai: 43%
	China	Ng	2008	Aged 18–49	5%	n/a	70%
	China	d’Arcangues	2011	Aged 18–38 Norplant users	Beijing: 4.6%	Beijing: 10.7%	Beijing: 84.7%
	India	Bhatt	2005	Ages not specified	10–20%^d^	20–60%^d^	20–70%^d^
	Indonesia	d’Arcangues	2011	Aged 18–38 Norplant users	Jakarta: 5.0%	Jakarta: 0%	Jakarta: 95%
Europe	Austria	Nappi	2016	Aged 18–45	32–34%^e^	27–31%^e^	37–39%^e^
	Belgium	Nappi	2016	Aged 18–45	33–54%^e^	15–30%^e^	31–37%^e^
	Czech Republic	Szarewski	2012	Aged 15–49^c^	17%	46%	n/a
	France	Szarewski	2012	Aged 15–49^c^	17%	38%	n/a
	France	Nappi	2016	Aged 18–45	38–53%^e^	15–19%^e^	32–43%^e^
	Germany	Wiegratz	2004	Aged 15–19, 25–34, 45–49, and 52–57	Women aged 15–57: 48%^b^ Women aged 15–49: 41%^b^	Women aged 15–57: 18%^b^ Women aged 15–49: 21%^b^	Women aged 15–57: 24%^b^ Women aged 15–49: 29%^b^
	Germany	Snow	2007	Aged 18–49	~ 8%^b^	~ 60%^b^	~ 30%^b^
	Germany	Szarewski	2012	Aged 15–49^c^	19%	42%	n/a
	Italy	Ferrero	2006	Mean age 36.6	29%	28%	44%
	Italy	Fruzzetti	2008	Aged 18 to 50	26%	42%	32%
	Italy	Szarewski	2012	Aged 15–49^c^	4%	42%	n/a
	Italy	Nappi	2016	Aged 18–45	17–18%^e^	30–33%^e^	49–53%^e^
	Poland	Nappi	2016	Aged 18–45	14–19%^e^	37–42%^e^	44% (no difference between use or nonuse of hormonal contraception)
	Spain	Nappi	2016	Aged 18–45	19–22%^e^	30–39%^e^	42–48%^e^
	Switzerland	Merki-Feld	2014	Aged 15–19, 25–34, and 45–49	30%	32%	37%
	United Kingdom	Glasier	2003	Aged 20+	Edinburgh: 37%	Edinburgh: 20%	Edinburgh: 33%
	United Kingdom	Szarewski	2012	Aged 15–49^c^	19%	52%	n/a

^a^Ranges by race and ethnicity

^b^Percentages were calculated or estimated from information presented in the articles

^c^Excluded women with no history of contraceptive use that would not consider future use

^d^Ranges by occupation and rural vs. urban location

^e^Ranges by use or non-use of hormonal contraception

Variation between studies (i.e., age, contraceptive history, relationship status, race/ethnicity, education, etc.) precludes disentangling the impact of each factor on women’s preferences, but some relationships were specifically assessed in individual studies. Greater preference for amenorrhea was generally observed in either the youngest [44, 55, 71] or the oldest groups of women surveyed [50, 64, 66, 69, 70, 72], while women in middle age categories (i.e., 24–34) appeared less accepting of amenorrhea [53, 60]. In contrast, no significant differences in preference for amenorrhea by age group were found in studies in Nigeria, South Africa, Scotland, Italy [55, 67]. Younger women were also generally more likely to desire less frequent (but non-amenorrheic) menstrual bleeding patterns [56, 60, 66, 68, 70]. A Swiss study found that while 37% of women preferred monthly bleeding, nearly as many (32%) preferred an interval of 2–6 months, with women aged 15–19 most likely to prefer two-monthly intervals [53]. Italian women reported no significant differences in preferences for other bleeding pattern lengths by age [67].

Three multi-country analyses showed that previous use of hormonal contraception was associated with increased willingness to consider non-standard bleeding patterns [56, 70, 73], though this was not observed in two European studies [66, 69]. Six studies in various regions described less interest in non-standard bleeding patterns among married or cohabitating women (as compared with unmarried, non-cohabitating, or divorced women) [56, 58, 64]. In the United States, black and/or Hispanic women were most likely to believe monthly menstruation is necessary [61]. In one study, white women reported being more open to amenorrhea than black women (49% vs. 29%), though authors noted a correlation between race and study site, preventing the differentiation of racial and regional differences [58]. In a South African study, more white women (29%) than black women (9%); reported a preference for amenorrhea over other bleeding patterns [55]. Other studies examined whether relationships existed between preference for amenorrhea (or other non-monthly bleeding patterns) and factors such as occupation [56, 63], parity and desire for more children [55, 66], religiosity [44, 55, 56, 64, 65], and women’s current bleeding characteristics [55, 58, 65, 73]; findings for each relationship varied by context, and in some cases, showed significant associations in different directions.

Some studies assessed preferences regarding menstrual regularity and flow (vs. bleeding intervals). Bleeding regularity and predictability emerged as a key preference in two multi-country studies [70, 74], while another multi-country study found that 58% women would accept temporary irregularity if it ultimately led to fewer bleeding episodes or amenorrhea over time (ranging from 34% of women in Russia to 76% of women in Brazil) [72]. Lighter menstruation was viewed as a contraceptive benefit in some studies [54, 58, 75].

### CIMBCs as a reason for non-use, dissatisfaction, or discontinuation

Seventy-one included studies assessed women’s discontinuation, dissatisfaction, or non-use of contraception due to experience or perception of CIMBCs (Additional file 1) [43–50, 52–54, 72, 74–132]. Most pertained to a specific contraceptive method (implants: 20, IUDs: 12, combined OCPs: 10, progestin-only and combined injectables: 4, vaginal ring: 2), while 13 addressed multiple methods and 10 were not method-specific. While bleeding changes may have been inconsistently defined (by researchers and study participants) across studies, spotting, unpredictable, frequent or irregular bleeding were defined as negative side effects in 42 studies [43, 48–50, 52–54, 72, 75, 77, 78, 81, 82, 84, 86, 88–92, 94, 97, 98, 100, 102, 105, 106, 108–111, 116–119, 122, 123, 127–129, 132], 22 studies noted that heavy or prolonged bleeding were poorly tolerated [49, 53, 54, 77, 83, 84, 91, 92, 98, 101, 103, 104, 106, 110, 111, 113–115, 123, 129, 131], and 22 studies found contraceptive-induced amenorrhea to be problematic [44, 48–50, 76, 78, 79, 84, 86, 90, 91, 98, 100–102, 106, 113, 115, 123].

#### Non-use

Ten studies (including seven qualitative studies) examined whether CIMBCs caused women to hesitate or decide not to use contraception [43, 45–47, 49, 74, 82, 87, 104, 106]. A cross-sectional study on long-acting reversible contraception (LARCs) in the UK reported that the potential for irregular bleeding disincentivized method use [82]. A systematic review on LARCs found that though various CIMBCs were perceived both positively and negatively, heavy or irregular bleeding were generally viewed negatively [49]. A large study in eight developed countries among women interested in combined hormonal methods found that small proportions (3–5%) did not choose the contraceptive pill, ring or patch due to the potential for the absence of regular bleeding [74]. Women in qualitative studies in the US and New Zealand were concerned about using amenorrhea-inducing methods because it would complicate knowing if they were pregnant [46, 106], or (in New Zealand and Turkey) because they viewed menstruation as normal and healthy [45, 106]. Various health concerns related to CIMBCs (detailed below), also impacted willingness to use contraception [43, 47, 87].

#### Dissatisfaction

Twenty-one studies addressed how CIMBCs impacted method satisfaction [48–50, 52, 53, 75, 76, 83, 84, 88, 90, 92, 97, 99, 105, 107–110, 114, 132]; 13 of these calculated estimates of bleeding-related reasons for dissatisfaction [53, 75, 76, 83, 84, 92, 105, 107–110, 114, 132]. We did not detect clear patterns in dissatisfaction for CIMBCs by geographic area, but several studies showed that despite dissatisfaction with specific aspects of a given method, some women may nonetheless choose to continue use.

Various methods induce different bleeding changes (i.e., injectables often induce amenorrhea, copper IUDs are associated with a temporary increase in heavy bleeding, etc.) [14, 16]. Menstrual abnormalities were the most common complaint among women using injectables in studies in Mexico and Nigeria [76, 84]. Among the 71% of Nigerian progestin-only injectable users who were dissatisfied with CIMBCs, amenorrhea was the most commonly disliked change (67% of those dissatisfied) [76]. Similar proportions of progestin-only (24%) and combined injectable (Cyclofem) (22%) users in Kenya described CIMBCs as their least liked method characteristic, despite the finding that women using progestin-only injectables were much more likely to experience amenorrhea (71% versus 21% in Cyclofem users) [107].

Among Nestorone implant users in Brazil, Chile and the Dominican Republic, the most common complaints were an increase in flow and duration of bleeding, as well as amenorrhea [108]. Similarly, half of method complaints in a retrospective medical chart review among Thai implant users were bleeding-related (prolonged bleeding, spotting, and amenorrhea) [114]. Irregular bleeding was the most commonly reported problem (22%) among Irish implant users [105]. A Kenyan study found 7–8% of IUD and implant users reported that their bleeding patterns were not acceptable [132]. A US study of IUD and implant use found that 17–19% of participants disliked heavy or prolonged bleeding while only 5% disliked lighter and decreased bleeding [92]. IUD users in Bangladesh most commonly reported heavy bleeding as an unwanted side effect [83]. Spotting between periods led 12% of hormonal IUD users in the US to report disliking the method, while another 13% disliked the IUD for factors which included other bleeding-related reasons [75]. Only 6% of women with hormonal IUD experience in Austria indicated that they were “really not satisfied” with their bleeding pattern [110]. Lastly, a clinical study comparing a standard and tailored use of OCPs (with the assumption of less bleeding with tailored regimens), found lighter bleeding to be among the most commonly reported side effect, but surprisingly, more women using a tailored regimen were dissatisfied with bleeding patterns (3% versus 11%) [109].

#### Switching and discontinuation

Sixty studies [44, 47, 48, 50, 52, 54, 72, 75–81, 83–91, 93–103, 105, 107–123, 125–131] reported at least one subject discontinuing or switching a contraceptive method specifically due to bleeding changes, and 40 measured the proportion of subjects doing so [48, 54, 72, 75–81, 83–86, 88, 89, 91, 93–96, 98, 100–102, 105, 107–110, 112–115, 121, 123, 125–128, 130]. Several included studies (40) found that CIMBCs were either the leading cause or among the top reasons for discontinuation [44, 48, 52, 54, 75–79, 83–87, 89, 91, 94–100, 102, 105, 107, 108, 110, 112, 113, 115, 121–123, 125–129, 131]. Three studies reported that between 0 and 10% of discontinuers did so due to CIMBCs [81, 112, 114], 9 reported 11–25% [48, 54, 88, 94, 108–110, 128, 130], 13 reported 26–50% [75, 77, 78, 80, 84, 89, 101, 102, 105, 109, 115, 121, 129] and 15 reported over 50% [54, 76, 79, 83–86, 95, 96, 98, 100, 121, 123, 125, 126]. Detailed information on studies assessing discontinuation according to specific contraceptive method is available in Additional file 2.

### Conceptual linkages between CIMBCs and health risks or side effects

Thirty-three studies had information pertinent to this topic, including six in Africa [43, 47, 87, 104, 107, 122], nine in the Americas [44, 46, 57, 103, 133–137], five in Asia [45, 63, 64, 83, 114], nine in Europe [51, 52, 60, 67, 88, 97, 109, 110, 138], two in Oceania [106, 139], and two in multi-country studies [50, 140]. In studies across multiple countries, including Mali, Kenya, South Africa, Brazil, Spain, the Dominican Republic, Canada, the US, and the UK, regular menstruation was viewed by many women as a marker of health and fertility, as well as providing reassurance of not being pregnant [47, 50, 51, 57, 87, 103, 133–135, 137, 138]. Women in South Africa, Mali, and Brazil, adolescents in South Africa and the US, rural housewives in India, and poor urban women in Turkey perceived that menstruation cleansed the body of “dirty blood” or toxins [44–47, 63, 87, 122].

However, associations between menstruation and health were not uniformly positive. Some women in South Africa and Mali perceived menstruation as positive but simultaneously dirty, inconvenient, or uncomfortable [47, 50, 87]. Chinese women reported needing to take an average of 3.3 sick days from work per year due to painful menstruation [64]. A higher percentage of Spanish women in one survey reported not liking anything about menstruation other than feeling it was natural and healthy [138]. Some CIMBCs were perceived as beneficial, for example, in Spain and Austria, women initiating LNG-IUD use reported appreciating reductions in heavy menstrual bleeding and painful periods [88, 110]. For some South African women, living with HIV raised anxieties about the need to protect family members from items soiled by their blood, as well as fears that not menstruating might “keep the HIV inside” of their bodies [47, 50]. In a multi-country study among women living with HIV the proportion who perceived amenorrhea as an ideal feature in a contraceptive method was generally low: 28% in Kenya, 22% in South Africa, and 0% in Brazil [50].

Contraceptive-induced amenorrhea raised health-related concerns in several settings. Young Malian women viewed amenorrhea as abnormal or indicative of illness [87]. Multiple study participants in South Africa and Ghana, as well as adolescents in the US, noted perceiving amenorrhea as “blocked” blood, and believed that if this blood did not exit the body, health issues (or even death) might ensue [43, 46, 47, 122]. Some South African adolescents also perceived that “blocked” blood eventually coming out too quickly could also lead to death [122]. A range of symptoms were understood as being caused by amenorrhea, including nosebleeds, blood clots, fibroids, bad skin, anorexia, weight gain, and more [44, 47, 104, 122]. Among adolescents in the US, amenorrhea (and irregular bleeding) also caused doubts about the effectiveness of their contraceptive method, and accompanying fears about being pregnant [46].

However, contraceptive-induced amenorrhea was not consistently perceived negatively. For example, some young abortion patients in New Zealand felt that it had both positive and negative aspects [106], and some students in India preferred it, so long as it didn’t interfere with their feminine looks [63]. In a Kenyan randomized trial comparing a progestin-only injectable (DMPA) to a combined injectable (Cyclofem), 71% of DMPA users and 12% of Cyclofem users were amenorrheic by 12 months. 78% of women in both groups said what they liked most about their method was the “lack of side effects” – suggesting that most did not view amenorrhea as a side effect [107].

In addition to amenorrhea, health concerns around heavy or prolonged bleeding emerged across several contexts [46, 104, 114]. For example, Bangladeshi women who discontinued an IUD due to heavy bleeding said they felt emotionally and physically unwell, were unable to participate in various activities, and described being in a “bloodless body” [83]. Some also said this evoked fears about uterine perforation and potential death [83]. Some Malian woman also linked heavy bleeding to the possibility of death, or other health issues such as cancer [87]. Similarly, among some women in the UK, prolonged or heavy bleeding signified bodily damage or a “body out of control” [51, 97].

A key theme, generally related to amenorrhea but sometimes to excessive bleeding, pertained to fears of becoming permanently infertile [46, 87]. For example, in South Africa, some women perceived that “blocked” blood (amenorrhea) would cause the womb to “get tired” or that excessive bleeding would lead to infertility [47, 122]. In Turkey, some women described fears that using contraception would cause their ovaries to get “lazy” [45]. However, some infertility fears were linked to the hormonal content of some contraceptives, rather than to bleeding changes [50].

Ten studies, primarily from higher-income countries, provided information specifically pertinent to how women perceive use of menstrual suppression in relation to health concerns [44, 52, 60, 67, 109, 133, 135, 136, 139, 140]. The largest of these was an online survey of over 4000 women across eight countries (Brazil, Canada, the Czech Republic, France, Germany, Italy, the UK, and the US) [140]. Health concerns were substantial for women with respect to menstrual suppression, with 42% of women believing that postponing monthly bleeding would have negative effects on their health [140].

### Women’s responses to deliberate menstrual suppression

Menstrual suppression involves using certain types of hormonal contraception in specific ways to deliberately avoid monthly bleeding, either on a short-term basis for specific life events (i.e., travel, honeymoon, athletic events, etc.) or on a longer-term basis to suppress menstruation for longer timeframes. Among 28 studies on menstrual suppression [44, 51–53, 57, 59–62, 64, 66–68, 70, 78, 93, 103, 109, 111, 127, 133–136, 138–141], many themes were similar to those described above, and when relevant, these studies are included in sections above. Given this, and since menstrual suppression represents the deliberate manipulation of the menstrual cycle (rather than as a “consequence” of standard contraceptive use, the main focus of this review), we address this topic only briefly.

The majority of studies focused on suppression through OCP use [57, 59–61, 66, 68, 70, 93, 103, 109, 111, 133–136, 138–141], with 6 to 65% of study participants reporting having suppressed menstruation [57, 59–61, 66, 68, 70, 133–136, 138–141]. Some studies discuss other hormonal methods [66, 78, 139] or use of hysterectomy to suppress menstruation [139]. Other considerations related to menstrual suppression included: practicality and convenience of avoiding menstruation [52, 59, 135, 139–141], fertility concerns [60, 61, 67, 134, 136, 139], perceptions of short and long term health effects [44, 51, 60, 61, 64, 67, 134, 136, 139, 140], cost of menstrual suppression [134] and feminine hygiene products [52, 135], impact of menstruation on activities [51, 59, 64, 66, 70, 135], management of pain, heavy bleeding or other undesirable menstrual symptoms [51, 53, 59, 60, 62, 135, 136, 141], concerns about becoming pregnant while suppressing menstruation [52, 60, 140], and information from or recommendation of a medical provider about menstrual suppression [57, 59, 61, 138].

### Other emergent themes

Several additional themes emerged. For example, multiple studies addressed how CIMBCs (or menstruation) positively or negatively impacted daily activities, including participation in domestic, work, school, sports, social, or religious life; sexual or emotional relationships; concentration or sleeping ability; or clothing choices and the need to manage excessive amounts of laundry (to wash fabrics used to absorb blood) [44, 50, 51, 56, 57, 63, 64, 66, 69, 70, 83, 87, 133, 137]. Bangladeshi women who discontinued IUD use due to perceived excessive menstrual bleeding described guilt for being unable while bleeding to pray or contribute to household tasks (e.g., tending cows or cooking) [83]. Some Indian women appreciated bleeding as it provided temporary relief from domestic chores [63], and in Brazil, an acceptable excuse to refuse sexual intercourse [44]. Several Malian women described how excessive bleeding increases concern that male partners may seek extramarital partnerships, as men are discouraged from sex with menstruating women [87]. In this context, non-pregnant amenorrheic women may be perceived as promiscuous, which can lead to social ostracization and divorce [87]. Given cultural prohibitions around participation in various activities during menstruation, CIMBCs can also “out” women attempting to use a method clandestinely [87, 106]. Furthermore, many of the studies reflecting these themes were conducted in low-resource settings, where menstrual hygiene products may be less accessible [142]. In a multi-country survey across eight largely higher-income countries, nearly one-third of women felt menstrual bleeding had a severe negative impact on their daily life, and most preferred to reduce bleeding frequency [70].

A related body of evidence measured favorable and unfavorable attitudes towards menstruation and associated factors [51, 53, 55, 58, 60, 62, 65, 68, 70, 103, 137]. For example, 62% of women in a Brazilian study [137] and 69% of women in a US study [58] noted disliking menstruation. Inconvenience and pain were common reasons [51, 58, 60, 65, 137], while feeling healthy, natural, womanly, or being reassured of not being pregnant were common themes for liking menstruation [45, 51, 55, 58, 60, 103, 137].

Providing information on potential or expected side effects, including CIMBCs, is a recommended component of comprehensive contraceptive counseling [143, 144]. While several studies indicated that at least some participants received some contraceptive counseling (prior to or during method use) on CIMBCs [45, 48, 72, 75, 87, 97, 98, 103, 113–115, 123, 124, 145] our search strategy identified few studies measuring the impact of counseling on method satisfaction or continuation. A few studies suggested that good contraceptive counseling may have improved method satisfaction or continuation rates, but none reported specific results to this effect [75, 103]. A study among LARC users in Brazil found no significant difference in discontinuation rates among women receiving “routine” versus “intensive” counseling including CIMBCs [124]. General family planning counseling (which may not have included appropriate bleeding-specific information) had no overall effect on discontinuation rates of IUD, implants and injectables among Egyptian women [115]. Among the implant users in the study, however, those experiencing longer bleeding lengths had a 2% increased hazards of discontinuation without counseling, and an 18% increased hazards of discontinuation with counseling; this seemingly counterintuitive result might relate to lack of adequate, method-specific counseling [115]. Also surprisingly, Dutch women specifically counseled on CIMBCs had lower 12- and 24-month implant continuation rates (72 and 53%, respectively) than previous similar studies [113]. Only one study included information about participants’ assessment of the quality of counseling they received on CIMBCs [75].

Some studies directly explored women’s perceptions of how bleeding patterns impacted their choice of contraception [51, 82], including tradeoffs between contraceptive effectiveness and CIMBCs [72]. For example, a survey administered in nine countries found that the percent of women who would consider using one of the most effective contraceptive methods, even if it were associated with menstrual cycle changes, ranged from 24% (in Italy) to 53% (in the UK and Brazil) though overall, younger women were less likely to consider this tradeoff [72]. Overall, 42% of women in that study would consider using one of the most effective contraceptive methods even when informed that their menstrual cycle would change and may become irregular [72]. Other studies examined which component of CIMBCs worried women [115] or the proportion of women who contacted health care providers to discuss bleeding concerns [85]. Finally, a few studies addressed impacts of contraception on menstrual-related issues (such as menstrual pain) [48, 101, 123], or used vignettes pertaining to women of different ages, relationships statuses, and life events, to examine how participants thought through various scenarios involving CIMBCs [43].

## Conclusions

Substantial variability exists in terms of how women respond to CIMBCs – including what they prefer and what they are willing to tolerate – and these responses are shaped by individual and social influences. For example, women’s stated preferences for amenorrhea ranged from 0 to 65% across included surveys. Contraceptive-induced amenorrhea may be viewed more positively in certain geographical regions (e.g., the Americas, some European and South American countries; though little comparative data is available in Africa) and by certain subpopulations (e.g., women younger than 24 or older than 34). In several multi-country surveys, prior use of hormonal contraception was associated with greater openness to non-monthly bleeding patterns. While several included studies suggest that CIMBCs do substantially impact contraceptive non-use, dissatisfaction, and discontinuation, most studies assessing this domain specifically evaluated discontinuation. Specific menstrual bleeding pattern preferences vary widely across contexts and sub-populations, but it is clear that CIMBCs can impact multiple aspects of women’s daily lives, including health-related perceptions, experiences, and fears, as well as participation in domestic, work, school, sports, social, religious, sexual, or other activities [146, 147]. Furthermore, several studies suggest that menstrual regularity (whether as part of normal menstruation or less frequent bleeding patterns) may be perceived positively [70, 74], and unexpected bleeding may be perceived negatively [43, 46, 48–54, 72, 75, 77, 78, 81, 82, 86, 88–92, 94, 97, 98, 100, 102, 105, 106, 108–111, 114, 116–119, 122, 123, 127–129, 132]. Monthly bleeding may relate to the reassurance of not being pregnant [51, 52, 55, 57, 58, 60, 140] and perceptions of continued fecundity [46, 47, 58, 60, 67, 87, 106, 134, 137, 139]. As such, women’s responses to CIMBCs (and the factors correlated with those responses) should be broadly recognized as a key issue in contraceptive research, counseling, and product development. A substantial proportion of relevant studies come from Europe, Northern America, and other higher-income settings, so studying these issues in other regions (e.g., Africa, Asia, and Oceania) is particularly needed, as results from these contexts may not generalize to lower-income settings.

This scoping review fills a key gap in the literature by mapping recent data on women’s responses and preferences to CIMBCs, and follows methodological guidance for conduct of scoping reviews [37]. Limitations of this review include searching a single database (PubMed) and the challenge of crafting a search strategy that is both specific and sensitive to such a broad topic of inquiry. We iteratively tested multiple search strategies, hand-searched reference lists of key studies, and consulted with an expert group to identify additional relevant articles. Crafting clear study inclusion criteria was also challenging, given the wide variety of pertinent study designs. To maximize comprehensiveness and feasibility while minimizing inclusion of irrelevant or minimally informative studies, we required that studies reference women’s responses to CIMBCs in the title and/or abstract; this may have influenced which studies were included. For example, among studies assessing contraceptive discontinuation, if CIMBCs were not a top reason (and thus not mentioned in the abstract), inclusion was less likely, which could mean that other reasons for discontinuation are underrepresented among our included studies. However, among included studies, we did attempt, where possible, to determine whether CIMBCs or other factors were the primary reasons for discontinuation (or other outcomes). While scoping reviews are intended to broadly map a domain in the literature, future systematic reviews assessing multiple reasons for contraceptive discontinuation could assess whether this approach to study inclusion impacted our findings. Finally, like all scoping reviews, we did not assess underlying study quality [38].

Several recommendations for contraceptive researchers, providers, and product developers emerge from this review. For example, in large, nationally representative surveys, inclusion of response options more specific than “side-effects” or “health concerns” pertaining to CIBMCs would enable more precise quantification of the association of CIMBCs with unmet need for family planning and contraceptive discontinuation. Longitudinal studies collecting information on bleeding patterns should adhere to guidelines used to classify bleeding patterns, to enhance comparability across studies [26, 148]. Collecting and controlling for key variables believed to influence responses to CIMBCs (i.e., age, prior contraceptive use, etc.) could also enhance comparability. In addition to disparities in geographic distribution of studies, several overall research gaps remain, including understanding how women’s knowledge of various physiological processes (i.e., menstruation, contraceptive mechanisms of action, etc.) impacts responses to bleeding patterns; the impact of contraceptive-induced amenorrhea or irregular bleeding on timing of pregnancy recognition and reproductive options; and linkages between CIMBCs and menstrual hygiene management. Researchers should adopt a neutral stance when asking women about menstrual preferences (e.g., avoid assuming that amenorrhea is viewed positively or negatively), and should be familiar with the range of instruments which have been used to investigate women’s responses to various menstrual-related issues (e.g., Menstrual Attitudes Questionnaire, Menstrual Distress Questionnaire, Attitudes towards Menstrual Suppression Instrument, Inconvenience Due to Women’s Monthly Bleeding instrument, etc.); consideration of using common, standardized measurements across studies may also be valuable.

Contraceptive providers should take women’s concerns about CIMBCs seriously and address them in a non-judgmental manner, as these changes may not be viewed merely as a minor side effect and, in some cases, may have profound impacts on multiple aspects of women’s lives. Given varied views on whether monthly bleeding is necessary for optimal health [135], providers should also be aware that some individuals may be skeptical about medical advice regarding what is “safe” or “normal”. Future work could help to clarify paradoxical findings [115] or investigate limited impacts of some counseling approaches [149]. Development of a method-specific tool to assist providers in counseling and treatment options around CIMBCs may be useful, particularly for contraceptive methods that result in variable bleeding patterns in different women [150]. Similarly, prospectively eliciting individual’s bleeding preferences could assist in helping them select a method most likely to suit their needs, and identification of factors that could help predict which side effects (including specific bleeding changes) a woman might expect to experience when initiating a contraceptive method may assist providers to better tailor contraceptive counseling [151]. Addressing some women’s concerns that menstrual irregularity is associated with reduced contraceptive effectiveness may be important [46]. Finally, providers and contraceptive users should be aware of treatment options for management of unwanted CIMBCs [152–155] (e.g., non-steroidal anti-inflammatory drugs, combined oral contraceptive pills, etc.), though more research is also needed to refine treatment options and improve bleeding patterns and user satisfaction/acceptability. Some evidence does suggest that treating undesirable CIMBCs may improve contraceptive continuation [156, 157].

Development of new contraceptive or MPT products hold promise from a public health perspective [158], but actual impact may be inhibited if acceptability (and consequently, adherence) is not adequately addressed [146, 159]. Studies on responses to CIMBCs within regions which would be targeted for rollout of new products may be useful during development stages, in order to enhance product acceptability. Furthermore, provision of clear information around expected CIMBCs for new products can help providers assist women to anticipate and manage these changes, and help avoid negative perceptions from becoming associated with new products. Ideally, product development will continue to expand method options to meet diverse women’s ideal contraceptive profiles (including preferred bleeding patterns), so contraceptors are not required to tolerate undesirable product characteristics in order to use effective pregnancy prevention strategies.

Overall, the importance of how women perceive and respond to CIMBCs may be currently underappreciated in the reproductive health field, despite likely – and potentially substantial – impacts on key issues such as contraceptive discontinuation and unmet need for modern contraception. Contraceptive researchers, providers, and product developers – in addition to policy-makers, service delivery suppliers, and funders – can use the body of knowledge summarized in this scoping review to better ensure that women and girls have a reliable supply of contraceptive (and MPT) options that align with their preferences and effectively prevent unintended pregnancies and other adverse outcomes.

## Additional files

Additional file 1: Summary of studies including information on contraceptive discontinuation, dissatisfaction or nonuse due to bleeding related side effects. (DOCX 29 kb)

Additional file 2: CIMBCs and discontinuation by specific method. (DOCX 19 kb)
