# Supplementary material for: There might be blood: a scoping review on women’s responses to contraceptive-induced menstrual bleeding changes
Source: Reprod Health. 2018 Jun 26;15:114. doi: 10.1186/s12978-018-0561-0 (PMC6020216; doi:10.1186/s12978-018-0561-0)
Supplement: Supplementary file 1 — Summary of studies including information on contraceptive discontinuation, dissatisfaction or nonuse due to bleeding related side effects. (DOCX 29 kb) [file 12978_2018_561_MOESM1_ESM.docx]

| **Additional File 1. Summary of studies including information on contraceptive discontinuation, dissatisfaction or nonuse due to bleeding related side effects** | | | | | | | | | | |
| --- | --- | --- | --- | --- | --- | --- | --- | --- | --- | --- |
| **Last name of first author** | **Publication year** | **Countr(ies) included in study** | **Contraceptive method** | **Included qualitative data** | **Examined bleeding-related side effects with respect to contraceptive:** | | | **Described negative bleeding related side effects, specifically:** | | |
|  |  |  |  |  | **discontinuation** | **dissatisfaction** | **nonuse** | **amenorrhea** | **prolonged / heavy** | **irregular / spotting*** |
|  |  |  |  |  |  |  |  |  |  |  |
| Harvey | 2009 | Australia | Implant |  | X |  |  |  |  |  |
| Riney | 2009 | Ireland | Implant |  | X | X |  |  |  | X |
| Flores | 2005 | Mexico | Implant |  | X |  |  |  | X | X |
| Teunissen | 2014 | Netherlands | Implant |  | X |  |  | X | X |  |
| Ezegwui | 2011 | Nigeria | Implant |  | X |  |  |  |  | X |
| Mutihir | 2010 | Nigeria | Implant |  | X |  |  | X |  | X |
| Arribas-Mir | 2009 | Spain | Implant |  | X |  |  |  | X | X |
| Thamkhantho | 2009 | Thailand | Implant |  | X | X |  |  | X |  |
| Yildizbas | 2008 | Turkey | Implant |  | X |  |  | X | X | X |
| Hoggart | 2013 | UK | Implant | X | X | X |  |  |  | X |
| Lakha | 2006 | UK | Implant |  | X |  |  | X |  | X |
| Jeffreys | 2012 | UK | Implant |  | X |  |  | X | X | X |
| Casey | 2011 | US | Implant |  | X |  |  |  |  |  |
| Casey | 2013 | US | Implant |  | X |  |  | X |  | X |
| Deokar | 2011 | US | Implant |  | X |  |  | X | X | X |
| Obijuru | 2016 | US | Implant |  | X |  |  | X |  |  |
| Sivin | 2003 | Brazil, Chile, Dominican Republic | Implant |  | X | X |  |  |  | X |
| Darney | 2009 | US, Chile and Europe and Asia* | Implant |  | X |  |  |  |  | X |
| Blumenthal | 2008 | Multiple (review) | Implant |  | X |  |  |  |  |  |
| Mansour | 2008 | Multiple (review) | Implant |  | X |  |  | X | X |  |

**Appendix Table 1. Continued**

| **Last name of first author** | **Publication year** | **Countr(ies) included in study** | **Contraceptive method** | **Included qualitative data** | **Examined bleeding-related side effects with respect to contraceptive:** | | | **Described negative bleeding related side effects, specifically:** | | |
| --- | --- | --- | --- | --- | --- | --- | --- | --- | --- | --- |
|  |  |  |  |  | **discontinuation** | **dissatisfaction** | **nonuse** | **amenorrhea** | **prolonged/ heavy** | **irregular / spotting*** |
|  |  |  |  |  |  |  |  |  |  |  |
| Nanda | 2014 | Dominican Republic | Oral contraceptive pill |  | X |  |  |  | X |  |
| Stephenson | 2013 | UK | Oral contraceptive pill |  | X | X |  |  |  | X |
| Graham | 2013 | UK | Oral contraceptive pill | X |  | X |  |  |  | X |
| Anderson | 2003 | US | Oral contraceptive pill |  | X |  |  |  |  |  |
| Sulak | 2002 | US | Oral contraceptive pill |  | X | X |  |  | X | X |
| Archer | 2006 | North America | Oral contraceptive pill |  | X |  |  |  |  |  |
| Van Vliet | 2006 | Multiple (Systematic reviews) | Oral contraceptive pill |  | X |  |  |  |  | X |
| Van Vliet | 2006 | Multiple (Systematic reviews) | Oral contraceptive pill |  | X |  |  |  |  | X |
| Van Vliet | 2011 | Multiple (Systematic reviews) | Oral contraceptive pill |  | X |  |  |  |  | X |
| Van Vliet | 2011 | Multiple (Systematic reviews) | Oral contraceptive pill |  | X |  |  |  |  | X |
| Barreiros | 2007 | Brazil | Vaginal ring |  | X |  |  | X |  | X |
| Weisberg | 2005 | Australia, Dominican Republic, Chile, US, Finland | Vaginal ring |  | X |  |  |  |  |  |
| Bortolotti de Mello Jacobucci | 2006 | Brazil | Injectable |  | X |  |  |  |  | X |
| Ruminjo | 2005 | Kenya | Injectable |  | X | X |  |  |  |  |
| Canto de Cetina | 2004 | Mexico | Injectable |  | X | X |  | X | X | X |
| Adeyami | 2012 | Nigeria | Injectable |  | X | X |  | X |  |  |

**Appendix Table 1. Continued**

| **Last name of first author** | **Publication year** | **Countr(ies) included in study** | **Contraceptive method** | **Included qualitative data** | **Examined bleeding-related side effects with respect to contraceptive:** | | | **Described negative bleeding related side effects, specifically:** | | |
| --- | --- | --- | --- | --- | --- | --- | --- | --- | --- | --- |
|  |  |  |  |  | **discontinuation** | **dissatisfaction** | **nonuse** | **amenorrhea** | **prolonged/ heavy** | **irregular / spotting*** |
|  |  |  |  |  |  |  |  |  |  |  |
| Baldaszti | 2003 | Austria | IUD (LNG) |  | X | X |  | X |  | X |
| Stoegerer-Hecher | 2012 | Austria | IUD (LNG) |  | X | X |  |  | X | X |
| Bradley | 2009 | Bangladesh | IUD (Copper) | X | X | X |  |  | X |  |
| Osei | 2005 | Ghana | IUD (Copper) | X |  |  | X |  | X |  |
| Jenabi | 2006 | Iran | IUD (Copper) |  | X | X |  |  |  |  |
| Bastianelli | 2011 | Italy | IUD (LNG) |  | X |  |  | X |  |  |
| Cristobal | 2016 | Spain | IUD (LNG) |  | X | X |  |  |  | X |
| Diedrich | 2015 | US | IUD (Copper & LNG) |  | X |  |  |  |  |  |
| Jensen | 2008 | US | IUD (LNG) |  | X |  |  |  |  |  |
| Teal | 2012 | US | IUD (Copper & LNG) |  | X |  |  |  |  |  |
| Bahamondes | 2015 | Brazil, Chile, Dominican Republic, Hungary, Thailand, Turkey, Zimbabwe | IUD (Copper, LNG & ENG) |  | X | X |  | X | X | X |
| Rowe | 2016 | China and 8 unspecified countries | IUD (Copper & LNG) |  | X |  |  | X | X |  |
| Weisberg | 2014 | Australia | Multiple: Implant, IUD (LNG ) |  | X |  |  |  | X | X |
| Wong | 2009 | Australia | Multiple: Implant, IUD (Copper/LNG) |  | X |  |  |  |  |  |
| Modesto | 2014 | Brazil | Multiple: Implant, IUD (Copper/LNG) |  | X |  |  |  |  |  |
| Tolley | 2005 | Egypt | Multiple: Implants, Injectables, IUD | X | X |  |  | X | X |  |
| Hubacher | 2015 | Kenya | Multiple: Implant, IUD (LNG) |  |  | X |  |  |  | X |
| Bracken | 2014 | UK | Multiple: Implants, Injectables, IUD |  |  |  | X |  |  | X |
| Clark | 2006 | US | Multiple: Implant, Injectable, Oral contraceptive pill, and others |  |  |  | X |  |  |  |
| Dickerson | 2013 | US | Multiple: Implant, IUD (Copper/LNG) |  | X | X |  | X | X | X |
| Diedrich | 2015 | US | Multiple: Implant, IUD |  |  | X |  |  | X | X |
| Apter | 2016 | Australia, Finland, France, Norway, Sweden, UK, | Multiple: Implant, IUD (LNG) |  | X |  |  |  | X | X |

**Appendix Table 1. Continued**

| **Last name of first author** | **Publication year** | **Countr(ies) included in study** | **Contraceptive method** | **Included qualitative data** | **Examined bleeding-related side effects with respect to contraceptive:** | | | **Described negative bleeding related side effects, specifically:** | | |
| --- | --- | --- | --- | --- | --- | --- | --- | --- | --- | --- |
|  |  |  |  |  | **discontinuation** | **dissatisfaction** | **nonuse** | **amenorrhea** | **prolonged/ heavy** | **irregular / spotting*** |
|  |  |  |  |  |  |  |  |  |  |  |
| Coombe | 2016 | Australia, US, New Zealand, Japan, Canada, Western Europe** | Multiple: LARCs |  |  | X | X | X | X | X |
| Egarter | 2013 | Austria, Belgium, Czech Republic, Slovakia, Netherlands, Poland, Sweden, Switzerland, Israel, Russia, Ukraine | Multiple: Contraceptive Patch, Oral contraceptive pill, Vaginal ring |  | X |  |  |  |  |  |
| Edelman | 2014 | Multiple (systematic reviews) | Multiple: Patch, OCP, ring |  | X |  |  |  |  |  |
| Estanislau do Amaral | 2005 | Brazil | Non-specific | X | X |  |  | X |  |  |
| Hindin | 2014 | Ghana | Non-specific | X |  |  | X |  |  | X |
| Castle | 2003 | Mali | Non-specific | X | X |  | X |  |  |  |
| Rose | 2011 | New Zealand | Non-specific | X |  |  | X | X | X | X |
| Laher | 2010 | South Africa | Non-specific | X | X |  | X |  |  |  |
| Wood | 2006 | South Africa | Non-specific | X | X |  |  |  |  | X |
| Merki-Feld | 2014 | Switzerland | Non-specific |  |  | X |  |  | X | X |
| Ay | 2007 | Turkey | Non-specific | X |  |  | X |  |  |  |
| Hooper | 2010 | US, UK, France, Germany, Spain, Italy, Brazil, Australia, Russia | Non-specific |  | X |  |  |  |  | X |
| Todd | 2011 | Brazil, Kenya, South Africa | Non-specific | X | X | X |  | X |  | X |
| TOTAL (71) |  |  |  | 13 | 60 | 22 | 10 | 22 | 22 | 42 |

*Irregular bleeding and spotting were not consistently defined in all studies reporting these as negative side effects

**Countries not specified
