# Supplementary material for: There might be blood: a scoping review on women’s responses to contraceptive-induced menstrual bleeding changes
Source: Reprod Health. 2018 Jun 26;15:114. doi: 10.1186/s12978-018-0561-0 (PMC6020216; doi:10.1186/s12978-018-0561-0)
Supplement: Supplementary file 2 — CIMBCs and discontinuation by specific method. (DOCX 19 kb) [file 12978_2018_561_MOESM2_ESM.docx]

**Additional File 2. CIMBCs and discontinuation by specific method**

Nineteen studies on implants found at least some discontinuation due to CIMBCs, often due to irregular bleeding, though “irregular” was variably defined [72,75,80,81,84,86,89–93,95–97,100,103,108,109,118]. Among the top reasons for Implanon discontinuation in three studies were erratic bleeding in the Netherlands, frequent or unpredictable bleeding in Scotland and prolonged bleeding in Mexico [90,95,108]. A review of Implanon clinical trials in 11 countries found that about one-third of discontinuers had the implant removed due to bleeding-related reason described by participants [96]. The most common reasons cited were frequent irregular bleeding and prolonged bleeding. Three studies on etonogestral implants in US found the top reason for discontinuation were for bleeding-related reasons [80,86,120]. No studies specifically mentioned amenorrhea as the top reason for implant removal, though eight studies found at least some women discontinuing for this reason [72,75,84,90,93,96,103,109].

Eight studies contained information about discontinuation pertaining to copper or levonoregestrel IUD use [43,70,74,78,83,94,105,107], three found heavy or prolonged bleeding as a top reason [78,99,105]. A study among Austrian women using levonorgestrel IUDs reported an overall high continuation rate (90% at three years), but spotting or amenorrhea were responsible for about one-fourth of discontinuations; similar findings were reported in Italy [43,74].

Four included studies reported some discontinuation of injectable methods for bleeding-related reasons [71,76,79,102]. Among the 22% of women with follow up information who discontinued progestin-only injectables in a Nigerian study, abnormal bleeding (primarily amenorrhea) was the top reason [71]. A randomized trial comparing progestin-only and combined injectables in Kenya found that nearly the same proportion of users discontinued due to bleeding-related reasons (13% and 14%, respectively), despite vastly different bleeding experiences among users of the two types of injectables [102]. One study among Mexican women found that some injectable discontinuers poorly tolerated amenorrhea, prolonged, or irregular bleeding [79]. A study among teenagers in Brazil reported one participant discontinuing injections due to irregular bleeding, but the study had a large loss to follow-up and reasons for discontinuation could not properly be assessed [76].

Eight studies on OCPs (largely focused on menstrual suppression), including four systematic reviews, found some discontinuation due to CIMBCs [47,98,104,106,111–114]. For example, separate studies in the US and UK found that among women using extended OCP regimens, breakthrough or unpredictable bleeding was the most common reason for switching to the standard regimen, and also caused some discontinuation [47,106]. A randomized controlled trial among women using cyclic and continuous OCP regimens found no association between bleeding patterns and discontinuation, and few discontinuers reported CIMBCs as the reason [98]. Four Cochrane reviews reported no differences in discontinuation due to intermenstrual bleeding between biphasic vs. monophasic, biphasic vs. triphasic, quadriphasic vs. monophasic, or triphasic vs. monophasic pills, despite the fact that in the latter comparison (triphasic vs. monophasic) favorable bleeding patterns were reported for triphasic pills [111–114].

Two studies included information on bleeding-related discontinuation of vaginal rings [73,115]. A five-country (Australia, Chile, Dominican Republic, Finland and US) clinical trial of a Nesterone/ethinyl estradiol vaginal ring found a low discontinuation rate for menstrual concerns (1.4 to 2.7 women per 100 users, depending on the hormone dose tested) [115]. Among the 17% of Brazilian women who discontinued an extended-regimen etonogestral/ethinyl ring, 38% did so due to irregular bleeding or amenorrhea [73].

Five studies examined bleeding-related discontinuation for multiple methods [49,85,88,110,116]; and four focused on LARCs [85,110,115,116]. A U.S. study on hormonal and copper IUDs and etonogestrel implants found an association with prolonged bleeding and discontinuation (particularly for implant users) [85] and in two Australian studies bleeding abnormalities were the top reasons for discontinuation of these LARC methods (constituting the reason for between one-quarter to more than half of discontinuations) [49,116]. Another LARC study in Egypt found heavy or prolonged bleeding to be a top reason (49%) for discontinuation of implants, injections and IUDs, and found amenorrhea to be a major reason (approximately one-third) among injectable users [110]. Each additional day of bleeding (as compared with number of bleeding days prior to use) independently increased the likelihood of discontinuation by 2% for the implant users, 3% for the IUD users and 4% for the injectable users [110].

Lastly, six studies (including five qualitative studies) reported findings on bleeding-related discontinuation but did not focus on specific method, rather contraceptive use in general [39,42,45,67,82,117]. A large community study among current contraceptive users in eight countries (Australia, Brazil, France, Germany, Italy, Russia, Spain, UK and US) found 19% of women switched hormonal methods due bleeding issues (ranging from 14% in Spain to 24% in Brazil) [67]. Two qualitative studies of young women in Mali and South Africa found CIMBCs and irregularities to be common reasons for discontinuation [82,117]. Two qualitative studies among HIV positive women in Brazil, Kenya and South Africa also found CIMBCs, including amenorrhea, to be major causes of discontinuation [42,45].
